# Portal Vein Variations, Clinical Correlation, and Embryological Explanation: A Review Article

**DOI:** 10.7759/cureus.36400

**Published:** 2023-03-20

**Authors:** Gareema Tyagi, Roshan K Jha

**Affiliations:** 1 Anatomy, Jawaharlal Nehru Medical College, Datta Meghe Institute of Medical Sciences, Wardha, IND; 2 Biochemistry, Jawaharlal Nehru Medical College, Datta Meghe Institute of Medical Sciences, Wardha, IND

**Keywords:** tips, portal hypertension, anatomic variations, venous system, portal vein

## Abstract

Portal vein (PV) is a large vein that collects blood from the abdominal part of gall bladder, pancreas, alimentary tract, and spleen and transports to the liver. One of the parts of the extraembryonic venous system, the vitelline veins, is where PV starts. In about five weeks of gestation, a venous plexus is formed, and variations in this plexus lead to portal variance. The junction of superior mesenteric and splenic veins is typically where the vein begins to network. There are five types of branching patterns of the right PV: conventional branching, trifurcation branching, early branching, separate segment 7 branching, and separate segment 6 branching. To perform pancreatic, duodenal, and liver surgeries, knowledge of variations in PV formation is important. For surgical and interventional operations to be accurate, it is crucial to understand the architecture of the PV and its anomalies. As distinct regions of the brain connect with one another, portal architecture is frequently observed in imaging investigations. Portal hypertension is characterized as an increase in blood pressure in the portal venous system (PVS) in the context of severe liver disease, such as cirrhosis. Non-invasive methods for examining the anatomy and anomalies of the PV include ultrasound, computed tomography (CT), and magnetic resonance (MR). There are many abnormalities of PVS that have been discussed in the articles such as Congenital PV Absence; PV Branches Congenitally Grow in Structure; Hypoplasia, Atresia, and Stenosis of the PV; and Portosystemic Shunts.

## Introduction and background

The portal vein (PV) transports blood to the liver. PV is the main blood vessel that essentially is the drainage of the superior mesenteric vein, which includes from the latter half of the second part of the duodenum all the way to the transverse colon and parts of the stomach, greater omentum, spleen, pancreas, and gallbladder in the abdominal region. In length, the PV is around 8 cm. The superior mesenteric and splenic veins converge posterior to the pancreas’ neck to form PV. The splenic vein runs directly from the splenic hilum to the pancreas, where it is located immediately posteriorly. The body and tail of the pancreas lie anterior to the splenic vein [1]. The superior mesenteric vein parallels the superior mesenteric artery's (SMA) course on its lateral side as it travels superiorly to merge with the splenic vein at the PV junction. The superior mesenteric vein is most easily observed with ultrasonography in longitudinal views [2]. The PV in the liver separates into sinusoids, which are then emptied via the hepatic vein and further into the inferior vena cava. It is known as the PV because its primary tributary, the superior mesenteric vein, starts in one set of capillaries (in the stomach) and ends in a different set of capillaries in the liver. The results of liver resections, transplantations, and interventional radiological procedures can be impacted by the prevalence of PV variants [3]. Knowledge of variations in PV formation is very helpful for surgeons performing pancreatic, duodenal, and liver surgeries [2].

## Review

Methodology

**Figure 1:**
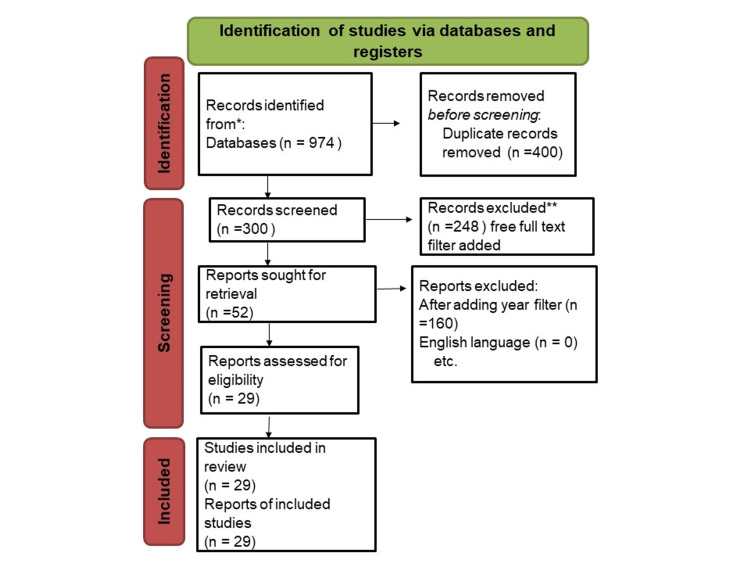
PRISMA flowchart of search. PRISMA: Preferred Reporting Items for Systematic Reviews and Meta-Analyses.

We undertook a systematic search through PubMed and CENTRAL in November 2020 using keywords such as "Portal vein variation" (PVV) and "embryological explanation" (EE) ((PVV [Title/Abstract]) OR (PVV (Title/Abstract]) OR (koch*[Title/Abstract)) OR ("PVV" [MeSH Terms]) AND ("EE" [Title/Abstract]) OR (EE [Title/ Abstract)) OR ("EE" [MeSH Terms)). We additionally searched for key references from bibliographies of the relevant studies. The search was updated in February 2022. One reviewer independently monitored the retrieved studies against the inclusion criteria, in the beginning, based on the title and abstract and then on full texts. Another reviewer also reviewed approximately 20% of these studies to validate the inclusion of studies (Figure 1).

Embryology

Embryology is the study of how embryos form, and one of the things that it can tell us is where the different veins in an embryo come from. The vitelline vein is one of these veins, and it gives rise to one of the three major veins in an embryo. The vitelline vein splits off into the PV, which develops into one of the body's major veins. The right and left vitelline veins unite about five weeks of gestation to create a venous plexus that encircles the duodenum. This venous plexus has three parts: two on the ventral side of the duodenum and one on the dorsal side of the duodenum. This plexus then terminates in the sinus venosus [4]. By 10 weeks, a part of this venous plexus selectively dissolves, forming the adult PV. The right vitelline vein forms the right PV, the left vitelline vein forms a ventral anastomosis with the left PV, and the left vitelline vein forms the main PV. Variations in the growth of the venous plexus and selective involution lead to PV variance [5].

Anatomic variations of the right PV

Five common branching patterns have been identified (Table 1) [5].

**Table 1:** Anatomic variations of the right PV. Image credit: Gareema Tyagi.

S. No.	Types	Name
1.	Type 1	Conventional branching
2.	Type 2	Trifurcation branching
3.	Type 3	Early branching
4.	Type 4	Separate segment 7 branching
5.	Type 5	Separate segment 6 branching

Type 1: Conventional Branching

In a typical branching pattern, the major PV is divided into branches on the right and left. Segments 6 and 7 of the liver are supplied by the posterior branch of the right PV, while segments 5 and 8 of the liver are supplied by the anterior branch. The right portal trunk is a sizable and reliable target for a transjugular intrahepatic portosystemic shunt (TIPS) due to type 1 anatomy. This shunt is anterior to and next to the right hepatic vein. This also enables acute angle puncture of the right PV trunk [6]. Obtuse angle shunts enable direct transhepatic advancement of devices (such as catheters, sheaths, and stents) from the right jugular vein. Because an obtuse angle is associated with shunt malfunction, maintaining a suitably acute shunt angle is essential to enhancing shunt input from the PV. Some suggestions can enhance portal decompression. The covered TIPS stent-graft component may not be able to restrict the posterior segment of the PV branch because this type of branching allows for selective puncture of the right anterior channel. This is noteworthy since a previous study revealed a link between possible hepatic ischemia and post-TIPS segmental PV thrombosis (PVT) [5].

Type 2: Trifurcation Branching

According to type 2 PV anatomy, the main PV is divided into a left PV, a right anterior PV that supplies segments 5 and 8 of the liver, and a right posterior PV that supplies segments 6 and 7 at the same location at the craniocaudal level. The creation of TIPS can be more difficult than usual because a vessel must be punctured into one of the right portal branches, either the right anterior branch or the posterior branch, which is often smaller than the target vessel in the right portal trunk [5]. The intrahepatic tract can be perforated on one side of the liver in a number of locations (right anterior, posterior, or circumferential), which may result in a steep shunt angle that makes moving the device more challenging. If the shunt is inadequately flowing, this could be a problem. It is possible to make sure that a section of the posterior part of the PV branch is not blocked by the underlying portion of the thrombosed interportal sinus by performing type 2 anatomy, which entails selective puncture of the right anterior portal vein, to increase consistency. Targeting the sectional portal venous branch can be substituted with a triple PV puncture [7].

Type 3: Early Branching

When the right posterior PV undergoes early branching, the first branch, or type 3 (early) branch, serves liver segments 6 and 7. Because the right anterior PV and left PV divide at the same level and the smaller right anterior or posterior PV puncture target may be harder to find and less predictable than the larger right main portal trunk target, creating TIPS in this type of branching may be more difficult [8]. Targeting smaller segmental target vessels, such as the right anterior or right posterior PV, would necessitate the use of a more peripheral approach in order to prevent acute shunt angulation. This could lead to anatomical structures similar to type 2. On the other hand, the right PV bifurcation may help in the development of TIPS by enabling the right anterior segmental vascular to be specifically punctured, theoretically improving portal flow to the right posterior segments and preventing hepatic ischemia. Puncturing the right posterior PV might require gaining access to the right hepatic vein from a more distant location [5].

Type 4: Separate Segment 7 Branching

In type 4 (separate segment 7) anatomy, the right PV is divided into two vessels: the smaller vessel supplying only to liver segment 7 and the bigger vessel portioning liver segments 5, 6, and 8, beyond the left PV originating from the main PV. Type 4 plants influence TIPS formation similarly to type 1 anatomy. With this option, a relevant right ingress trunk can be placed where it would typically be closer and inferior to the right hepatic vein. In addition, similar to type 1 branching plants, if the proper main hole or door in the vessel container is targeted, a blunt change in angulation may be accomplished through the transhepatic area, expediting the inline tool advancement area and optimizing change inflow for ingress decompression [5].

Type 5: Separate Segment 6 Branching

In type 5 (separate segment 6) anatomy, the right PV divides into two vessels: one that solely supplies liver segment 6 and another arm that extends past the beginning of the abandoned ingress tone to supply liver divisions 5, 7, and 8. Type 5 plants can assist in the development of TIPS, same as type 1 branching and type 4 branching. This option permits a large right entrance core that is specifically positioned anteriorly and inferiorly to the right hepatic tone in a specific area. By aiming for a proper ingress body, it is possible to more accurately direct designs below the transhepatic tract, prevent doorway occlusion, and still maximize flow through the change [5].

Abnormalities of portal venous system 

Congenital PV Absence 

The superior mesenteric vein and splenic vein circulate blood around the liver and empty into a systemic vein when the PV is congenitally absent [9]. The liver is entirely disregarded as all portal blood from type I shunts is sent to systemic veins (e.g., congenital absence of PV). There are many potential health problems that can result from having the PV congenitally missing. This kind of shunt is often referred to as a complete or side shunt. Only a minor fraction of the portal venous flow is sent through the liver by a type II shunt. It is also known as a "partial" or "side/side" shunt [10]. A congenital absence of the PV is frequently associated with other congenital disorders, such as cardiac malformations, skeletal problems (such as Goldenhar syndrome), situs inversus, polysplenia, and liver problems (such as localized nodular hyperplasia, hepatocellular carcinoma, hepatoblastoma, and adenoma, as well as biliary atresia, portal hypertension (PH), and hepatic encephalopathy) [11].

PV Branches Congenitally Grow in Structure

One congenital defect that is frequently documented is congenital agenesis of the major portal branches. It is important to understand the difference between atrophy caused by an illness and atrophy that is congenitally present. The removal of a particular branch of the portal system may be followed by the absence of a particular liver lobe, even if the portal supply to a usually normal liver can be maintained by variable portal morphology. It is possible that thrombosis inside the portal branch that supplies the injured region during embryological maturation may be the cause of the removal of a portal branch and its associated lobe of the liver [12].

Hypoplasia, Atresia, and Stenosis of the PV

A hypoplastic entrance vein may be a common symptom in people with biliary atresia, and it has been associated with greater problems following liver transplantation in terms of both overall survival and thrombosis. A blockage may result in concomitant hypertension, splenomegaly, and gastrointestinal bleeding as well as PV hypoplasia, atresia, and stenosis. The name of this condition is Banti syndrome [13].

Portosystemic Shunts

Without going through the liver, a portosystemic shunt diverts blood from the portal venous system (PVS) to the systemic vein. Extrahepatic and intrahepatic shunts are the two basic varieties. One instance of an extrahepatic portosystemic shunt is congenital lack of the PV [14].

Surgical implications

It is clinically essential to be aware of the variations in the PV before surgery in order to precisely locate liver lesions, select donors for liver transplants, remove tumors, perform PV embolization (PVE), and perform TIPS because the PV, along with hepatic veins, determines the segmental anatomy (TIPS). Preprocedural cross-sectional imaging would provide a full grasp of the anatomy of the PV variations, which would significantly lower the likelihood of unanticipated surgical difficulties due to the rise in percutaneous hepatobiliary interventions and difficult surgical resections [15].

The TIPS technique, which is based on the blind canalization of the PV by a puncture that comes from the hepatic vein, entails the implantation of a stent that connects the PV to the hepatic vein. A successful TIPS should be created between the right hepatic vein and the right PV. The normal type 1 PV anatomy's predictable placement of the PV in relation to the hepatic vein is responsible for the procedure's high success rates [16]. Therefore, having a detailed understanding of the anatomical variations of PV is essential for the creation of TIPS. Due to the clinical consequences of type 2 and type 3 variants, there might not be one primary, bigger right trunk present; hence, the final target might be smaller in caliber. Therefore, cross-sectional imaging both before and during the TIPS treatment to assess the venous anatomy is advised in order to assure a high success rate and avoid issues [17].

Prior to major hepatectomy, the PVE, a modern vascular intervention procedure, is carried out to enlarge the liver that the surgeon left in place before the procedure. To do this, the liver branches that must ultimately be removed four weeks before surgery are embolized [18].

Hepatobiliary surgeons normally seek at least 25% (in the event of a baseline healthy liver) and 40% as the future liver remnant (FLR) following hepatectomy (in the case of a diseased liver). When the expected FLR is low, PVE is utilized to try to increase FLR volume [18]. Therefore, a suitable FLR is always required for a successful big hepatectomy. Therefore, the PVE is the most reliable, secure, and efficient method for causing sufficient hepatic hypertrophy to sustain the FLR during a planned liver resection [19].

Portal hypertension

Increased portal vascular resistance, increased portal venous blood flow, or a combination of both can lead to PH. In veterinary medicine, portal pressure is rarely measured, so the diagnosis of PH is done by identifying complications such as ascites, multiple acquired portosystemic shunts, and hepatic encephalopathy. Additionally, managing these outcomes is the main objective of PH treatment [20]. PH is characterized as an increase in blood pressure in the PVS in the context of severe liver disease, such as cirrhosis. The onset of PH is one of the most important adverse outcomes of chronic liver disease. Even if a patient has not experienced any symptoms in years, they can still be deemed compensated. Hepatic decompensation is defined by PH's overt clinical presentation, though. With a markedly detrimental effect on patients' life expectancy and quality, the development of hepatic decompensation marks a significant turning point in the course of advanced liver disease [21].

PH affects the extrahepatic vascular beds, resulting in collateral vessel development and arterial vasodilation in the splanchnic and systemic circulations. Increased blood flow to the PV as a result of this worsens PH and finally leads to the hyperdynamic circulatory syndrome. As a result, ascites or esophageal varices appear [22].

Symptoms

 Blood in vomit, blood in stools, and a bloated stomach.

Diagnosis

Blood tests can reveal signs of kidney and liver dysfunction. Overactiveness of the spleen in removing WBCs can be determined from a complete blood count test. Hence, many blood disorders can be known that may cause PH [23]. Duplex Doppler ultrasound is usually suggested as the first test. It helps in the diagnosis of cirrhosis and PH. It gives a detailed image of PVS [24]. Endoscopy is also suitable for examining any signs of active or recent bleeding [23].

Treatment of PH

Beta-blockers help lower blood pressure (such as propranolol). Lactulose is a synthetic sugar that is being used by doctors to treat symptoms of hepatic encephalopathy. Antibiotics are used for treating bacterial infections (such as rifaximin).

Clinical Aspects 

**Figure 2:**
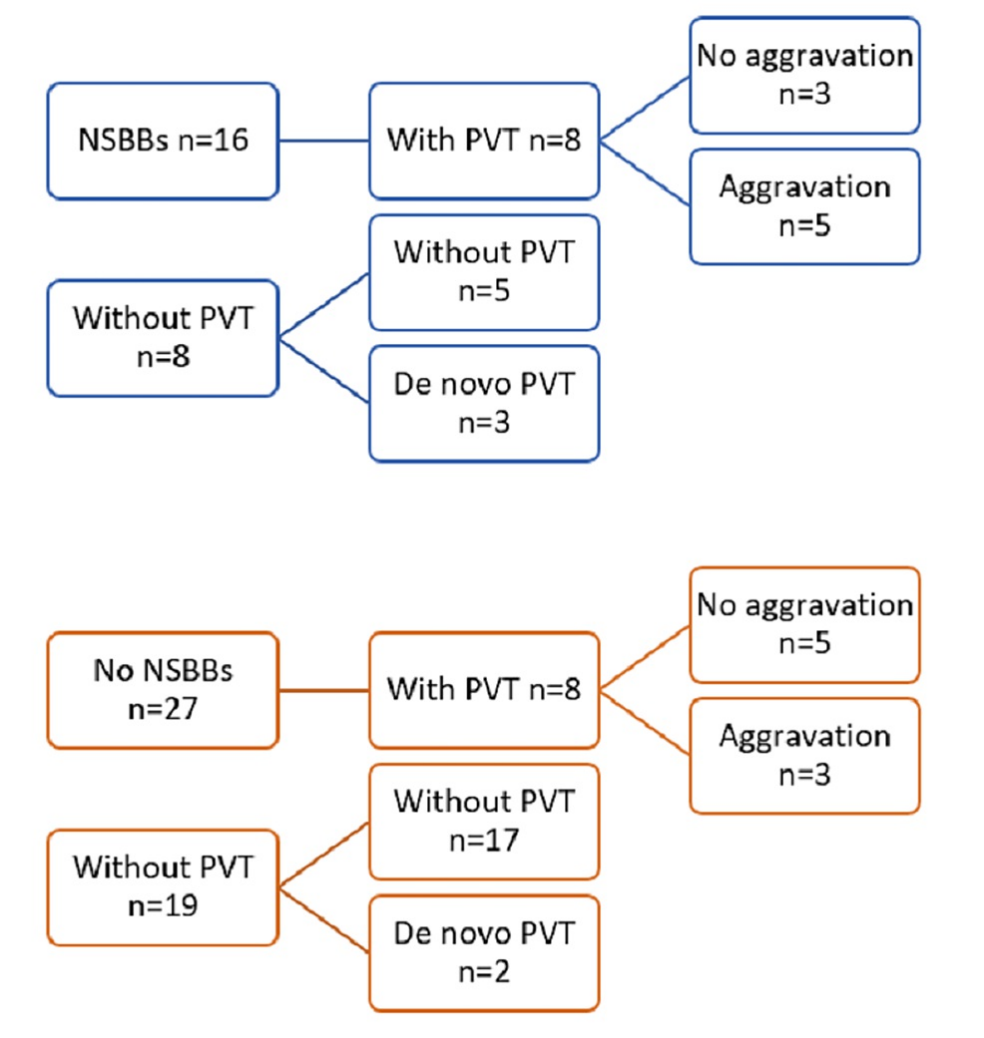
Flowchart of course of PVT. PVT: portal vein thrombosis; NSBBs: non-selective beta-blockers. Image credit: Gareema Tyagi.

PVT is a common complication of cirrhosis, especially in the advanced stages of the disease. PVT risk factors include slow blood flow, vessel wall damage, and hypercoagulability, all of which are symptoms of advanced cirrhosis. Because of the impaired hepatic synthesis of both pro- and anticoagulants, hemostasis is rebalanced, making it more prone to thrombosis or even bleeding [25]. The standard treatment for PVT in cirrhosis is vitamin K antagonist or low molecular weight heparin. With the introduction of new direct-acting oral anticoagulants (DOACs), there is a paradigm shift toward using DOACs for the treatment of PVT in cirrhotic patients [26]. DOACs are useful for certain diseases by reducing the burden of subcutaneous injections and the need for frequent monitoring with regular blood tests to ensure adequate therapeutic doses. With the development of PVT, cirrhosis can occur. PVT can exacerbate PH and cause liver decompensation. International guidelines recommend at least three months of anticoagulant therapy for all patients with acute PVT [27]. Cirrhosis can occur with the development of PVT. PVT can exacerbate PH and cause liver decompensation. International guidelines recommend at least three months of anticoagulant therapy for all patients with acute PVT (Figure 2) [28,29].

## Conclusions

PV is a large vein that collects blood from the abdominal region of the digestive tract, spleen, gall bladder, and pancreas and transports it to the liver. It is formed by the union of the superior mesenteric and splenic veins, which are located posterior to the pancreatic neck. PV anomalies, which are rather frequent, can affect the outcomes of liver resections, transplants, and interventional radiological procedures. The diversity of PV results from variations in the rate of expansion of this venous plexus and from selective involution. Five common branching patterns have been observed in the right PV: conventional, trifurcation, early, separate segment 7, and separate segment 6. When the PV is congenitally missing, the blood delivered by the superior mesenteric vein and splenic vein skips the liver and empties into the systemic circulation. Congenital agenesis of the major portal branches is one congenital abnormality that is frequently reported. It is critical to recognize the distinction between atrophy brought on by a disease and atrophy that is a congenital feature. Patients with biliary atresia may frequently develop a hypoplastic entrance vein, which has been associated with more complications after liver transplantation, such as lower survival and thrombosis. A "portosystemic shunt" is a technique used to redirect blood from the PV to the systemic vein. TIPS is a procedure that inserts a stent to connect the PV and hepatic vein. It is based on a puncture made from the hepatic vein that canalizes the PV blindly. A successful TIPS should develop between the right hepatic vein and PV on the right. Therefore, successful development of TIPS requires a thorough study of the anatomical differences of PV. PH can be brought on by an increase in portal vascular resistance, portal venous blood flow, or both (PH). Ascites, many acquired portosystemic shunts, and hepatic encephalopathy are common diagnoses since portal pressure is rarely directly assessed in veterinary practice.
